# Validation of angiography-based FFR in non-culprit vessels of patients presenting with STEMI

**DOI:** 10.1007/s00392-025-02729-x

**Published:** 2025-09-08

**Authors:** Jari A. van der Eijk, Frederik T. W. Groenland, Alessandra Scoccia, Annemieke C. Ziedses des Plantes, Jager Huang, Rutger-Jan Nuis, Jeroen M. Wilschut, Wijnand K. den Dekker, Roberto Diletti, Isabella Kardys, Mariusz Tomaniak, Nicolas M. Van Mieghem, Joost Daemen

**Affiliations:** 1 Department of (Interventional) Cardiology, Thoraxcenter, Erasmus University Medical Center, Room Rg-628, P.O. Box 2040, 3000 CA Rotterdam, the Netherlands; 2 Department of Cardiology, Medical University of Warsaw, Warsaw, Poland

**Keywords:** Acute coronary syndrome, Fractional flow reserve, Percutaneous coronary intervention, ST elevation myocardial infarction

## Abstract

**Background:**

Fractional flow reserve (FFR) for non-culprit lesions (NCLs) in patients with ST-elevation myocardial infarction (STEMI) can be influenced by temporary changes in microvascular resistance. Angiography-derived vessel fractional flow reserve (vFFR) has been tested as a less-invasive alternative.

**Aims:**

The FAST STEMI II study aimed to assess the diagnostic performance of acute-setting vFFR vs. FFR for intermediate NCLs in STEMI patients.

**Methods:**

FAST STEMI II is a prospective two-center cohort study including STEMI patients with ≥ 1 intermediate NCL (50–90% diameter stenosis). Patients with cardiogenic shock, prior revascularization of the non-culprit vessel, or aorta-ostial lesions were excluded. Following primary percutaneous coronary intervention (PCI), vFFR, FFR, resting full-cycle ratio (RFR), coronary flow reserve (CFR), and index of microcirculatory resistance (IMR) measurements of the NCL were performed.

**Results:**

A total of 111 patients were included. Median [25th–75th percentile] vFFR and FFR were 0.83 [0.74–0.88] and 0.83 [0.80–0.90], respectively. vFFR had a moderate to good discriminative ability to predict FFR ≤ 0.80 (AUC: 0.78; 95% CI: 0.68–0.89; p < 0.001) with a moderate correlation (r = 0.54; p < 0.001). Diagnostic accuracy, sensitivity, specificity, positive predictive value, and negative predictive value of vFFR to predict FFR ≤ 0.80 were 72%, 76%, 70%, 53%, and 87%, respectively. Microvascular dysfunction (CFR < 2.0 and IMR ≥ 25) was observed in 33 (31%) patients. In patients with microvascular dysfunction, median vFFR and FFR values were 0.76 [0.71–0.86] and 0.85 [0.77–0.90], respectively (p = 0.002).

**Conclusions:**

We found moderate correlation between vFFR and FFR in NCLs of patients undergoing primary PCI. Discordance between vFFR and FFR was associated with the presence of microvascular dysfunction.

The study was conducted in accordance with Good Clinical Practice and the Declaration of Helsinki and was registered at 22-jun-2023 on clinicaltrials.gov under the identifier NCT05698719.

**Graphical Abstract:**

**Figure:**
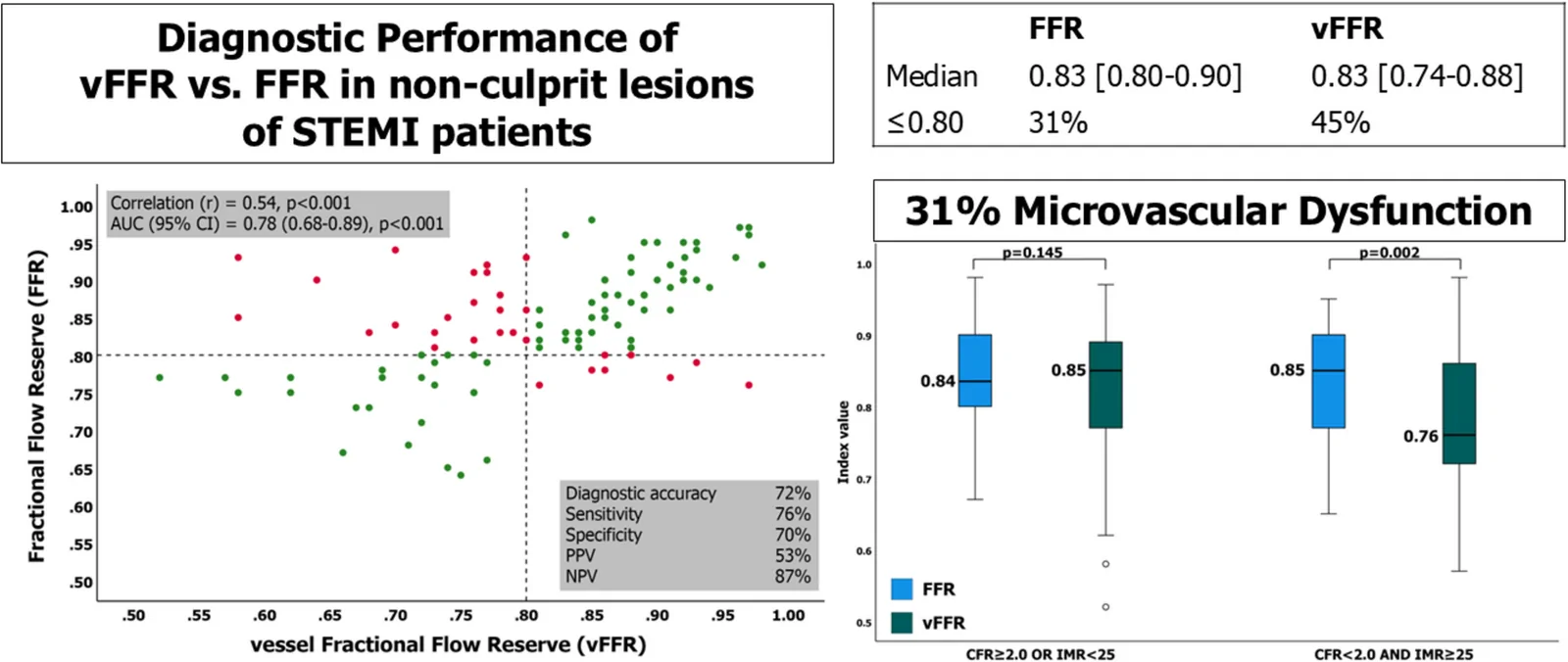

**Supplementary Information:**

The online version contains supplementary material available at https://doi.org/10.1007/s00392-025-02729-x.

## Introduction

In patients presenting with ST-segment elevation myocardial infarction (STEMI), multivessel disease (MVD) with intermediate to significant coronary lesions in non-infarct-related arteries is observed in up to 50% of cases [1]. Complete revascularization in these patients is associated with improved clinical outcomes [2, 3].

Fractional flow reserve (FFR) and non-hyperemic pressure ratios (NHPRs) are increasingly used to guide revascularization with a Class IA recommendation in contemporary revascularization guidelines [4, 5]. While most evidence on the use of physiology-guided percutaneous coronary intervention (PCI) is based on studies in patients presenting with either stable or unstable coronary artery disease, there is conflicting evidence on the use of FFR to guide complete revascularization in patients presenting with STEMI [6–10]. Data on the role of NHPRs in this particular setting are scarce, and discordance between FFR and NHPRs has been linked to the presence of microvascular dysfunction and may occur in up to 22% of cases [11–13].

Angiography-derived NHPR, such as vessel fractional flow reserve (vFFR), has been validated as an accurate alternative to FFR that may likely enhance the adoption of physiology-guided complete revascularization in daily practice [14–16]. vFFR estimates FFR using three-dimensional (3D) reconstructed coronary anatomy and computational fluid dynamics, without requiring hyperemic agents or pressure wires. However, limited data is available on the diagnostic accuracy of NHPR in patients presenting with STEMI. The aim of this study was to assess the diagnostic performance of vFFR relative to FFR, performed directly after successful primary PCI in the interrogation of intermediate non-culprit lesions (NCLs) in STEMI patients.

## Methods

### Study design and study population

FAST STEMI II is a prospective two-center international observational cohort study. Consecutive adult patients presenting with STEMI undergoing primary PCI with at least one intermediate NCL (50–90% diameter stenosis, visually estimated) were considered eligible for this study. Exclusion criteria were cardiac arrest or cardiogenic shock, prior revascularization of the non-culprit vessel, or aorta-ostial disease (diameter stenosis > 50%) in the non-culprit vessel. Additional exclusion criteria were excessive overlap, foreshortening, or tortuosity, precluding vFFR computation. In case of multiple non-infarct-related arteries with intermediate lesions, the non-infarct-related artery including the lesion with the most severe diameter stenosis, based on 3D quantitative coronary angiography (QCA), was used for the analysis of the primary and secondary outcome measures.

The ethical committees of each participating site approved the study protocol. The study was conducted in accordance with Good Clinical Practice and the Declaration of Helsinki and was registered on clinicaltrials.gov under the identifier NCT05698719. All patients provided written informed consent.

### Study procedures and study materials

All procedures were performed according to standard clinical practice. During the index procedure, vFFR, resting full-cycle ratio (RFR), thermodilution-based coronary flow reserve (CFR), index of microcirculatory resistance (IMR), and FFR measurements (CoroVentis CoroFlow System, Abbott Vascular, Santa Clara, United States of America) were consecutively performed with the position of the pressure wire at least 2 cm distal to the lesion of interest in case of vessel calibre > 2.25 mm. vFFR was also calculated offline by a single experienced researcher who was blinded to the acute-setting measurements (JVDE). The vFFR analyses were performed on two orthogonal views to create a 3D reconstruction of the coronary arteries with CAAS Workstation 8.5 (Pie Medical Imaging, Maastricht, Netherlands) in accordance with previously validated methodology [14].

### Study endpoints

The primary endpoint was the diagnostic performance of vFFR for the physiological assessment of intermediate NCLs in STEMI patients, with FFR as the reference standard. Secondary endpoints included: 1) the correlation and diagnostic performance of acute-setting vFFR with RFR and offline vFFR as the reference standard, 2) the correlation and diagnostic performance of offline vFFR with FFR and RFR as the reference standard, 3) the correlation and diagnostic performance of RFR with FFR as the reference standard, and 4) the relation between microvascular dysfunction, defined as CFR < 2.0 and IMR ≥ 25, and FFR, vFFR, and RFR. Diagnostic performance includes area under the curve (AUC), sensitivity, specificity, diagnostic accuracy, positive predictive value (PPV) and negative predictive value (NPV). Cutoff values were vFFR ≤ 0.80, FFR ≤ 0.80, and RFR ≤ 0.89. For thermodilution-based measurements, the cutoff values were CFR < 2.0, and IMR ≥ 25 [17].

### Sample size

Sample size calculations were performed based on previous trials investigating MVD in STEMI and the results of the FAST II study [14]. We expected 50% positive FFR measurements, and a diagnostic accuracy of 90% for vFFR, with the FFR as the reference standard. A sample size of 111 patients was needed to demonstrate this diagnostic accuracy with a 95% confidence interval (CI) width of 0.12 (0.83–0.95). Based on this sample size, we expected to demonstrate a sensitivity of 81% with a 95% CI width of 0.23 (0.69–0.91) and a specificity of 95% with a 95% CI width of 0.14 (0.85–0.99).

### Statistical analysis

The normality of the distributions of continuous variables was evaluated using the Shapiro–Wilk test. Normally distributed variables were expressed as mean ± standard deviation, while non-normally distributed variables were expressed as median with 25th – 75th percentile. Categorical variables were presented as counts with percentages. Continuous variables were compared within subgroups using the Wilcoxon signed-rank test or paired t-test, and between subgroups using the Mann Whitney U test or unpaired t-test, based on the distribution of the data. The difference in proportions between groups was tested using the Chi-squared test. The correlations between the different physiological indices were quantified using the Spearman’s correlation coefficient (r). The relation between vFFR, FFR, and RFR was displayed in scatter plots, while agreement was investigated with Bland–Altman plots, with corresponding 95% limits of agreement. Receiver operating characteristics curves were plotted with subsequent AUC calculation. The diagnostic performance of vFFR to identify FFR-significant lesions was assessed, including diagnostic accuracy, sensitivity, specificity, PPV and NPV with 95% CI. Statistical analyses were performed using SPSS statistical package, Version 28 (IBM Corp.). Two-sided p-values < 0.05 were considered statistically significant.

## Results

### Study population, baseline and procedural characteristics

A total of 111 patients were included in two European sites (Erasmus University Medical Center, Rotterdam, the Netherlands and Medical University Clinical Hospital, Warsaw, Poland) between July 2022 and December 2023. Among these patients, vFFR was successfully computed in 110 patients (99%), with one exclusion due to technical difficulties precluding vFFR computation. Similarly, RFR was available in 108 patients (97%), offline vFFR in 109 patients (98%), and both CFR and IMR in 105 patients (95%). Baseline and procedural characteristics are presented in Tables 1 and 2. Mean age was 63 ± 11 years and 71% of the patients were male. Diabetes was present in 14% of cases. The left anterior descending artery (LAD) was the non-culprit vessel in 59%. The median [25th – 75th percentile] vFFR was 0.83 [0.74–0.88], FFR was 0.83 [0.80–0.90], RFR was 0.90 [0.84–0.95], and offline vFFR was 0.83 [0.75–0.88]. The median [25th – 75th percentile] for CFR and IMR were 2.0 [1.5–2.7] and 24 [17–37], respectively. In total, vFFR was ≤ 0.80 in 45% of NCLs, compared to FFR ≤ 0.80 in 31% and RFR ≤ 0.89 in 48%.

**Table 1:** Baseline characteristics

Variable	N = 111
Age (years)	63 ± 11
Male sex	79 (71%)
Heart rate (bpm)	76 ± 15
Systolic blood pressure (mmHg)	121 ± 25
Diastolic blood pressure (mmHg)	73 ± 13
Cardiovascular risk factors	
Hypertension	52 (47%)
Dyslipidemia	36 (32%)
Diabetes	15 (14%)
Current smoker	38 (34%)
Family with CAD	49 (44%)
Medical history and comorbidity	
Prior PCI	10 (9%)
Prior MI	8 (7%)
Prior CVI/TIA	4 (4%)
Prior PAD	3 (3%)
GFR (mL/min/1.73 m^2^)	79 [63–90]
Haemoglobin (mmol/L)	8.6 ± 0.9
Culprit lesion location	
RCA	50 (45%)
LAD	32 (29%)
LCX	29 (26%)
PCI culprit lesion	111 (100%)

Values are presented as mean ± SD, median [25th – 75th percentile] or n (%). Legend: *CAD*, coronary artery disease; *CVI*, cerebrovascular incident; *GFR*, glomerular filtration rate; *LAD*, left anterior descending artery; *LCX*, left circumflex artery; *MI*, myocardial infarction; *PAD*, peripheral artery disease; *PCI*, percutaneous coronary intervention; *RCA*, right coronary artery; *SD*, standard deviation; *TIA*, transient ischemic attack

**Table 2:** Procedural characteristics

Non-culprit lesion location and characteristics	N = 111
LAD	66 (59%)
LCX	24 (22%)
RCA	21 (19%)
Focal disease	27 (24%)
Long lesion (> 28 mm)	44 (40%)
Bifurcation lesion	31 (28%)
Moderate or severe calcification	17 (15%)
Acute-setting 3D QCA (N = 107)	
Minimal lumen diameter (mm)	1.5 [1.3–1.9]
Diameter stenosis (%)	46 ± 10
Reference vessel diameter (mm)	2.9 [2.5–3.3]
Minimal lumen area (mm^2^)	1.8 [1.3–2.8]
Area stenosis (%)	70 ± 11
Reference vessel area (mm^2^)	6.5 [5.0–8.5]
Lesion length (mm)	19 [13–34]
Contour correction (%)	9 [5–12]
Indices	
vFFR, (N = 110)	0.81 ± 0.10; 0.83 [0.74–0.88]
FFR, (N = 111)	0.84 ± 0.07; 0.83 [0.80–0.90]
RFR, (N = 108)	0.89 ± 0.08; 0.90 [0.84–0.95]
Offline vFFR, (N = 109)	0.81 ± 0.09; 0.83 [0.75–0.88]
CFR, (N = 105)	2.3 ± 1.1; 2.0 [1.5–2.7]
IMR, (N = 105)	28 ± 16; 24 [17–37]
vFFR ≤ 0.80	49/110 (45%)
FFR ≤ 0.80	34/111 (31%)
RFR ≤ 0.89	52/108 (48%)
Offline vFFR ≤ 0.80	44/109 (40%)
CFR < 2.0	49/105 (47%)
IMR ≥ 25	52/105 (50%)
Microvascular dysfunction (CFR < 2.0 and IMR ≥ 25)	33/105 (31%)
PCI non-culprit lesion	38/111 (34%)
Direct PCI	22/38 (58%)
Staged PCI	16/38 (42%)
Stenting	38/38 (100%)
Total stent length (mm)	44.6 ± 19.4
Maximal stent diameter (mm)	3.0 [3.0–3.5]

Values are presented as mean ± SD, median [25th – 75th percentile] or n (%). Legend: *3D*, three-dimensional; *CFR*, coronary flow reserve; *FFR*, fractional flow reserve; *IMR*, index of microcirculatory resistance; *LAD*, left anterior descending artery; *LCX*, left circumflex artery; *PCI*, percutaneous coronary intervention; *QCA*, quantitative coronary angiography; *RCA*, right coronary artery; *RFR*, resting full-cycle ratio; *SD*, standard deviation; *vFFR*, vessel fractional flow reserve

### Correlation and diagnostic performance

**Fig. 1:**
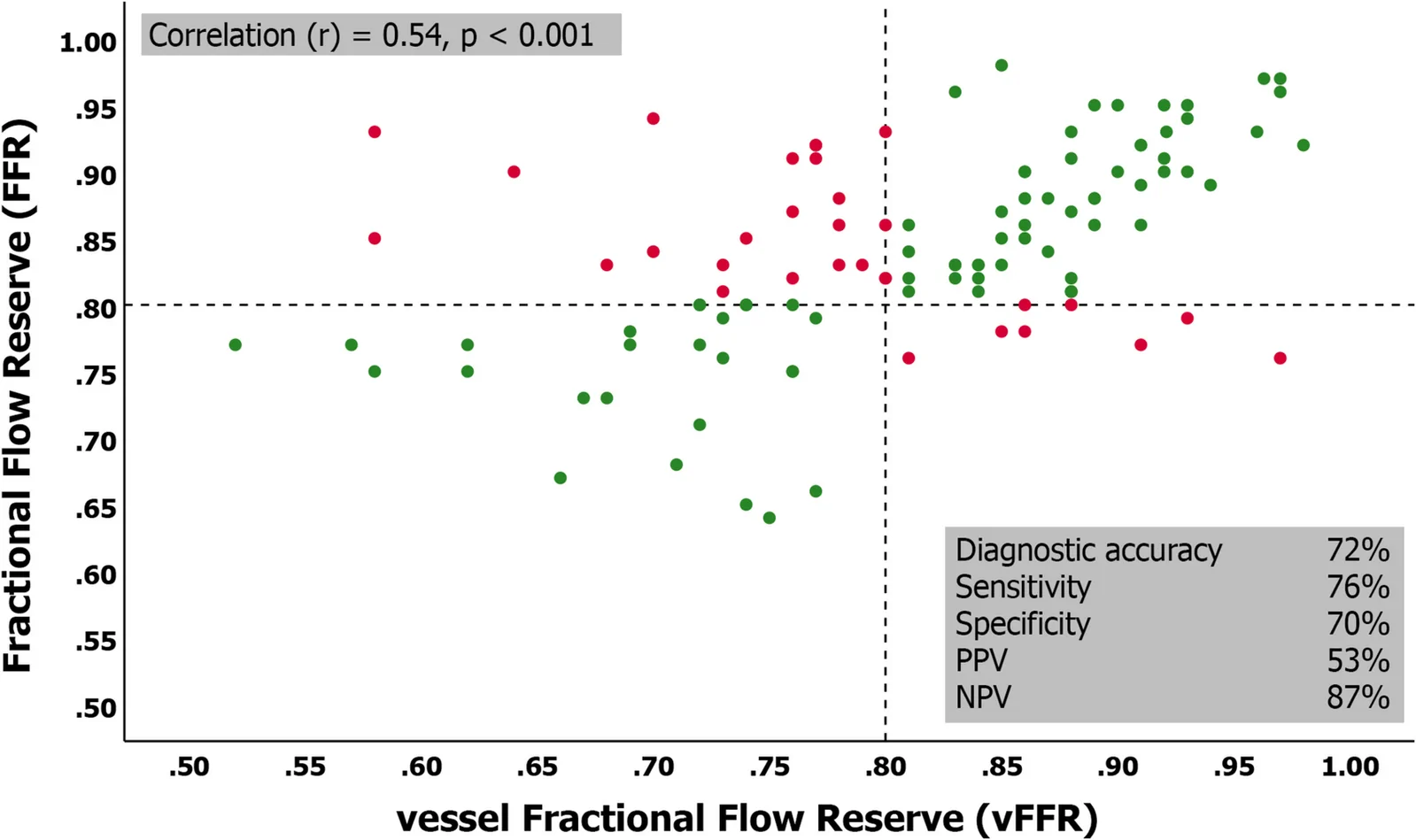
Scatter plot showing the relationship between vFFR vs. FFR (r = 0.54; p < 0.001). The green dots indicate concordance between indices, while the red dots indicate discordance between indices (based on the cutoff value of ≤ 0.80 for vFFR and FFR). The grey box below displays the diagnostic performance of vFFR with FFR as the reference standard. *FFR*, fractional flow reserve; *NPV*, negative predictive value; *PPV*, positive predictive value; *vFFR*, vessel fractional flow reserve

A moderate correlation (r = 0.54; p < 0.001) was found between vFFR and FFR (Fig. 1) with a median [25th – 75th percentile] difference of -0.02 [-0.08 – 0.02] (Fig. S1). vFFR had a moderate to good discriminative ability for FFR ≤ 0.80 (AUC: 0.78; 95% CI: 0.68–0.89; p < 0.001) (Fig. S2). Diagnostic accuracy, sensitivity, specificity, PPV, and NPV of vFFR ≤ 0.80 with FFR ≤ 0.80 as the reference standard were 72%, 76%,70%, 53%, and 87%, respectively (Fig. 1). Discordance between vFFR- and FFR-defined lesion significance was observed in 31 patients (28%), primarily driven by cases with vFFR ≤ 0.80 but FFR > 0.80 (23/31, 74%) (Fig. 1).

**Fig. 2:**
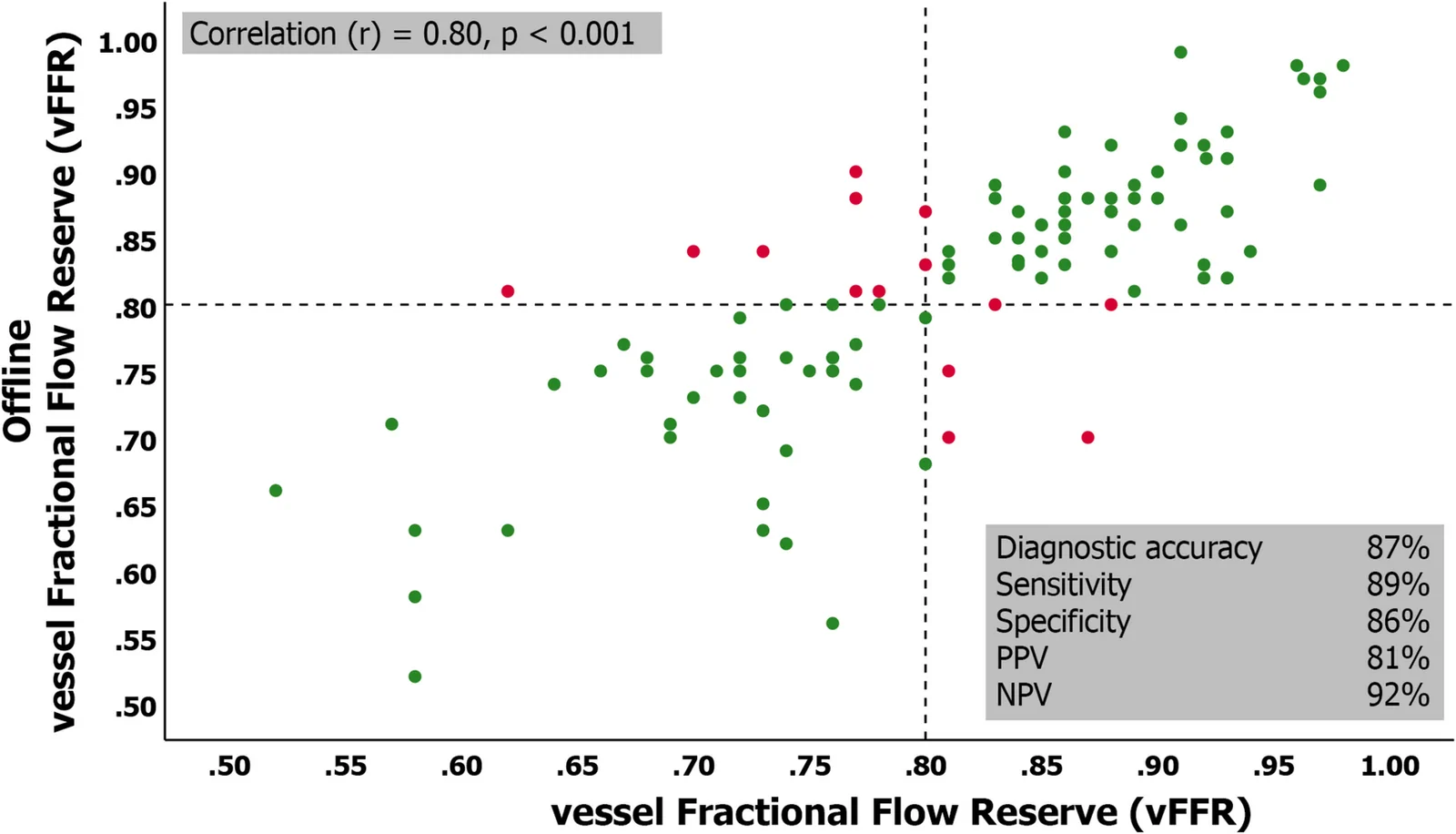
Scatter plot showing the relationship between acute-setting vFFR vs. offline vFFR (r = 0.80; p < 0.001). The green dots indicate concordance between indices, while the red dots indicate discordance between indices (based on the cutoff value of ≤ 0.80 for vFFR). The grey box below displays the diagnostic accuracy of acute-setting vFFR with offline vFFR as the reference standard. *NPV*, negative predictive value; *PPV*, positive predictive value; *vFFR*, vessel fractional flow reserve

Similar findings were observed for offline vFFR as compared to FFR with a moderate correlation (r = 0.54; p < 0.001) (Table S1), a moderate to good discriminative ability (AUC: 0.80; 95% CI: 0.72–0.89, p < 0.001) (Fig. S2), and a comparable diagnostic performance to predict FFR-significant lesions as acute-setting vFFR (Table S2). Furthermore, a strong correlation (r = 0.80; p < 0.001) was found between acute-setting and offline vFFR with a diagnostic accuracy of 87% (Fig. 2).

vFFR had a moderate to good discriminative ability for RFR ≤ 0.89 (AUC: 0.76; 95% CI: 0.66–0.85; p < 0.001) (Table S2). Diagnostic accuracy, sensitivity, specificity, PPV, and NPV of vFFR ≤ 0.80 with RFR as the reference standard were 72%, 67%, 76%, 73%, and 71%, respectively. Discordance between vFFR- and RFR-defined lesion significance was observed in 30 patients (28%), including 57% (17/30) cases with vFFR > 0.80 but RFR ≤ 0.89.

Similar to acute-setting and offline vFFR, RFR had a moderate to good discriminative ability for FFR ≤ 0.80 (AUC: 0.80; 95% CI: 0.71–0.89; p < 0.001) (Fig. S2). A full overview including the correlation and diagnostic performance for all different physiological indices is provided separately (Tables S1 and S2).

### Microvascular assessment

**Fig. 3:**
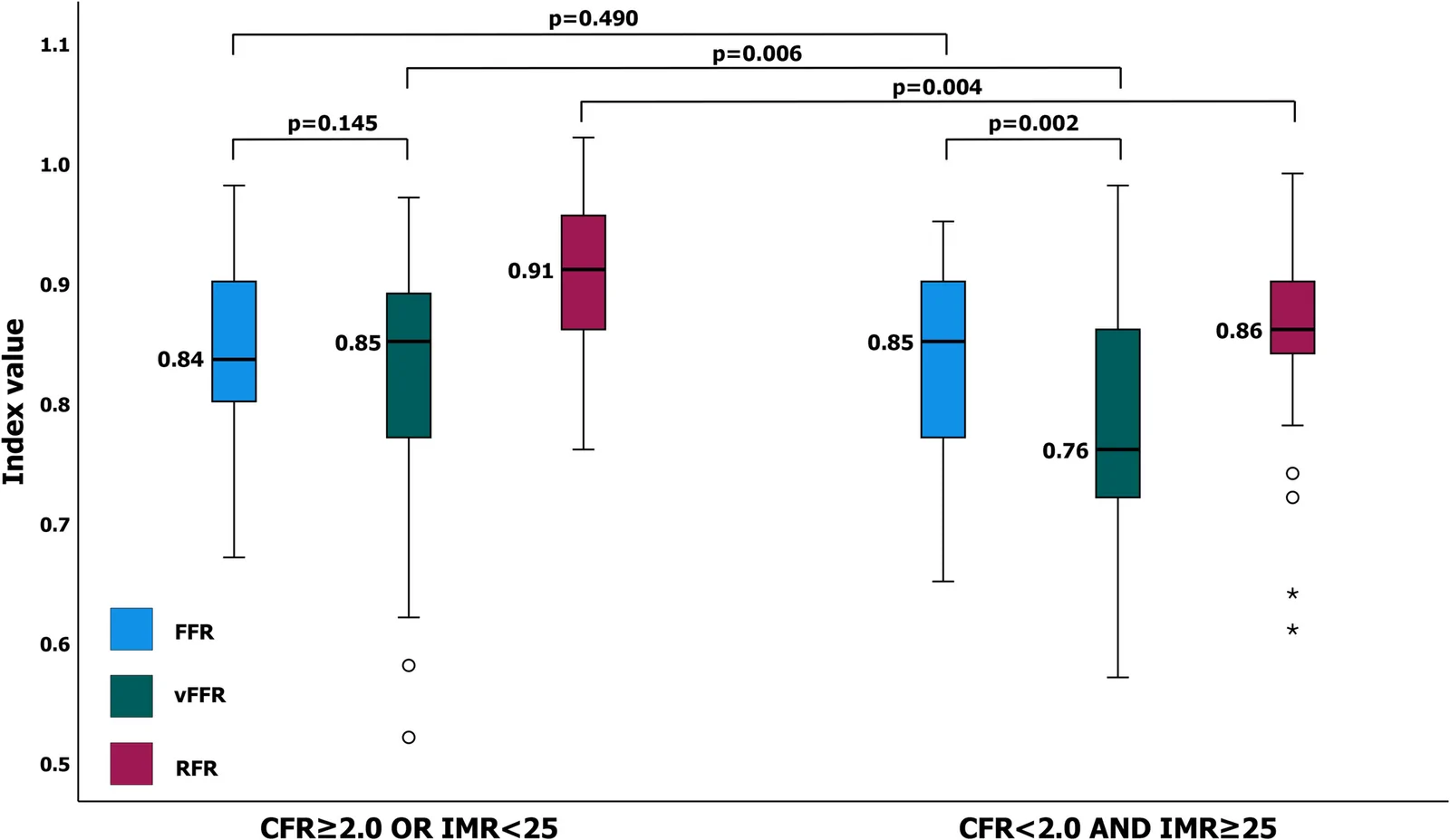
Comparison of vFFR, FFR, and RFR measurements in patients with and without microvascular dysfunction (CFR < 2.0 and IMR ≥ 25). Boxplots show the distribution of vFFR, FFR, and RFR values, with medians and p-values for group comparisons. *CFR*, coronary flow reserve; *FFR*, fractional flow reserve; *IMR*, index of microcirculatory resistance; *RFR*, resting full-cycle ratio; *vFFR*, vessel fractional flow reserve

Microvascular dysfunction was present in 33 patients (31%). In patients with microvascular dysfunction, median [25th – 75th percentile] vFFR was 0.76 [0.71–0.86], FFR was 0.85 [0.77–0.90], and RFR was 0.86 [0.84–0.91], while in patients without microvascular dysfunction, median [25th – 75th percentile] vFFR was 0.85 [0.77–0.89], FFR was 0.84 [0.80–0.90], and RFR was 0.91 [0.86–0.96] (Fig. 3). Median vFFR was significantly lower as compared to FFR in patients with microvascular dysfunction (p = 0.002), but not in patients without microvascular dysfunction (p = 0.145) (Fig. 3). The median [25th – 75th percentile] vFFR-FFR difference in patients with vs. without microvascular dysfunction was -0.05 [-0.11 – -0.01] vs. -0.01 [-0.06 – 0.03] (p = 0.006). Consistent results were found in a sensitivity analysis using CFR < 2.5 and IMR ≥ 25 (Fig. S3).

In patients with structural microvascular dysfunction (CFR < 2.0 and IMR ≥ 25), the median difference between vFFR and FFR was -0.05, while the median difference between vFFR and FFR was 0.01 in patients with functional microvascular dysfunction (CFR < 2.0 and IMR < 25). These findings may suggest that structural microvascular dysfunction had a negative impact on the correlation between vFFR and FFR.

## Discussion

The FAST STEMI II study was a prospective, international, two-center, observational study demonstrating the diagnostic performance of vFFR in identifying pressure-wire-based FFR-significant intermediate NCLs in STEMI patients. The main findings of this study can be summarized as follows: 1) a moderate to good diagnostic performance with a moderate correlation was found between vFFR and FFR, 2) microvascular dysfunction was diagnosed in 31% of cases, and 3) discordance between vFFR and FFR was present in 28% of cases and was associated with the presence of microvascular dysfunction.

Following several previous retrospective studies assessing the diagnostic accuracy of offline quantitative angiography-based physiology using the quantitative flow ratio (QFR) index (Medis Medical Imaging, Leiden, the Netherlands), the present study represents the first prospective validation of vFFR as compared to conventional wire-based physiology for NCL of STEMI patients undergoing primary PCI. Given the lack of consistent evidence supporting the use of physiology to guide NCL revascularization in a primary PCI setting, the assessment of the potential value of vFFR is of interest. Whereas conventional pressure-wire-based physiology in a primary PCI setting is hampered by temporary changes in microvascular function, the hyperemic response as used in the vFFR computational model is based on the theoretical increase of flow and thereby proved to remain stable between acute and staged settings [18, 19].

We identified the presence of microvascular dysfunction in 31% of cases, which may have contributed to the significantly lower values of vFFR as compared to FFR. As expected, in a primary PCI setting, the latter resulted in increased discordance between vFFR and FFR as compared to previous validation work performed in chronic coronary syndromes, unstable angina or non-ST-elevation acute coronary syndrome (NSTEMI) setting [14]. The latter is in line with previous validation studies on the performance of QFR in patients presenting with STEMI, which also found a lower diagnostic performance when using FFR as a reference [20].

Of interest, we found significantly lower vFFR values in patients with microvascular dysfunction as compared to patients without microvascular dysfunction. Albeit not significant, higher diameter stenosis, lower reference vessel diameter, combined with the significant lower minimal lumen diameter and minimal lumen area, suggest a more advanced disease state in this subgroup (Table S3). This difference could be explained by the significant older age of patients with microvascular dysfunction (Table S4). The impact of microvascular dysfunction was further illustrated by the higher percentage of RFR as opposed to FFR positive lesions, a phenomenon that has been previously described and explained by the fact that resting indices may tend to overestimate acute-setting lesion significance due to transient elevations in resting coronary flow in STEMI [19].

### Perspectives

Our findings may have several implications. As compared to FFR, vFFR may be a promising alternative to help guiding NCL revascularization in a primary PCI setting. Given the conflicting evidence supporting the use of conventional wire-based physiology in the FRAME-AMI and FLOWER MI study, multiple angiography-based FFR (sub)studies demonstrated promising findings [7, 9]. As such, Lee et al. reported higher event rates post NCL PCI in case of high QFR as compared to lesions that were deferred [21]. In addition, in the recent retrospective FAST STEMI I study, the highest event rates were observed in vFFR-positive non-revascularized NCLs [16]. These findings suggest that vFFR may provide added sensitivity in identifying lesions at risk, particularly where traditional FFR assessment may underestimate their significance. That said, future evidence is needed to establish the definitive role of vFFR to guide NCL revascularization in patients presenting with STEMI.

Despite their potential benefits, the use of angiography-based physiology indices, with QFR in particular, was recently challenged by the findings of the FAVOR-III EUROPE trial which discouraged the use of QFR to guide clinical decision making in a stable setting due to a 2.5% absolute increase in MACE rates as compared to a conventional pressure-wire-based FFR strategy [22]. An unanticipated finding in clear contrast to the numerous validation studies and the FAVOR-III China trial that identified QFR as an alternative to FFR and a superior metric to guide PCI as compared to angiography alone [23]. The eagerly awaited results of the ongoing multicenter randomized FAST III trial (NCT04931771) will address whether the use of vFFR will yield differential outcomes.

A potential explanation for the negative findings of FAVOR III EUROPE trial was the lower than anticipated performance of acute-setting vs. offline QFR – a concern that was already raised in the QREP study in which the inter- and intra-observer reproducibility for QFR computed from the same angiograms ranged from high to poor among multiple observers from different sites with an average agreement of 0.01 ± 0.08 for repeated measurements [24]. The reproducibility was dependent on the observer, angiographic quality and the stenosis severity as assessed by FFR. A finding that was not observed in the FAST I and previous corelab analyses of the FAST II studies using vFFR as the index of interest [14, 25, 26]. In the present study, we were able to extend these findings by demonstrating a strong correlation (r = 0.80) between acute-setting and offline vFFR, supporting the use of vFFR to enhance clinical decision making in the acute setting and prevent unnecessary staged procedures.

### Study limitations

This study has several limitations. First, the diagnostic performance measures turned out to be lower than anticipated in the sample size calculation. A larger sample size might be needed to provide more precise estimates of the diagnostic performance. Furthermore, as flow characteristics may differ between vessels, most interrogated lesions in the present study were located in the LAD. The importance of the latter was recently demonstrated in the FAVOR III EUROPE trial, in which the discordance in the number of lesions treated appeared largely driven by lesions in the left circumflex artery, which were significantly more often revascularized in the QFR- vs. the FFR-guided arm [22]. Finally, the effect of vFFR guidance on clinical outcomes was not studied and remain unclear. Dedicated studies are needed to assess the value of angiography-based physiology guided PCI of NCL in STEMI patients. The ongoing FAVOR V trial (NCT05669222) will compare the long-term clinical outcomes of the angiography-derived μFR-guided PCI strategy to standard treatment strategy in approximately 5000 patients presenting with STEMI and MVD.

### Conclusions

We found moderate correlation between vFFR and FFR in NCLs of patients undergoing primary PCI for STEMI. Discordance between vFFR and FFR was associated with the presence of microvascular dysfunction.

## Supplementary Information

Below is the link to the electronic supplementary material.

Supplementary file1 (DOCX 332 KB)

## Abbreviations

3D: Three-dimensional
AUC: Area under the curve
CFR: Coronary flow reserve
CI: Confidence interval
FFR: Fractional flow reserve
IMR: Index of microcirculatory resistance
LAD: Left anterior descending artery
MVD: Multivessel disease
NCL: Non-culprit lesion
NHPR: Non-hyperemic pressure ratio
NPV: Negative predictive value
PCI: Percutaneous coronary intervention
PPV: Positive predictive value
QCA: Quantitative coronary angiography
QFR: Quantitative flow ratio
RFR: Resting full-cycle ratio
STEMI: ST-segment elevation myocardial infarction
vFFR: Vessel fractional flow reserve
