# Supplementary material for: Validation of angiography-based FFR in non-culprit vessels of patients presenting with STEMI
Source: Clin Res Cardiol. 2025 Sep 8;115(10):1775–84. doi: 10.1007/s00392-025-02729-x (PMC13562354; doi:10.1007/s00392-025-02729-x)
Supplement: Supplementary file 1 [file 392_2025_2729_MOESM1_ESM.docx]

**Supplemental material**

*Supplemental Table 1. Correlation between physiology indices*

| Spearman’s rho | FFR | vFFR | Offline vFFR |
| --- | --- | --- | --- |
| vFFR | 0.54 (N=110)* | - |  |
| Offline vFFR | 0.54 (N=109)* | 0.80 (N=108)* | - |
| RFR | 0.67 (N=108)* | 0.50 (N=107)* | 0.52 (n=108)* |

Legend: FFR = fractional flow reserve, RFR = resting full-cycle ratio, vFFR = vessel fractional flow reserve. *p<0.001

*Supplemental Table 2. Diagnostic performance of physiology indices*

| **Diagnostic performance with FFR as reference standard.** | | | |
| --- | --- | --- | --- |
|  | vFFR (N=110) | RFR (N=108) | Offline vFFR (N=109) |
| AUC (95% CI);  p-value | 0.78 (0.68 – 0.89);  p<0.001 | 0.80 (0.71 – 0.89);  p<0.001 | 0.80 (0.72 – 0.89);  p<0.001 |
| Diagnostic accuracy (95% CI) | 72% (62% – 80%) | 69% (59% – 77%) | 74% (65% – 82%) |
| Sensitivity (95% CI) | 76% (59% – 89%) | 76% (59% – 89%) | 74% (56% – 87%) |
| Specificity (95% CI) | 70% (58% – 80%) | 65% (53% – 76%) | 75% (63% – 84%) |
| PPV (95% CI) | 53% (43% – 63%) | 50% (41% – 59%) | 57% (46% – 67%) |
| NPV (95% CI) | 87% (78% – 93%) | 86% (76% – 92%) | 86% (78% – 92%) |
| **Diagnostic performance with RFR as reference standard.** | | | |
|  | FFR (N=108) | vFFR (N=107) | Offline vFFR (N=108) |
| AUC (95% CI);  p-value | 0.80 (0.72 – 0.88);  p<0.001 | 0.76 (0.66 – 0.85);  p<0.001 | 0.79 (0.70 – 0.87);  p<0.001 |
| Diagnostic accuracy (95% CI) | 69% (59% – 77%) | 72% (62% – 80%) | 72% (63% – 80%) |
| Sensitivity (95% CI) | 50% (36% – 64%) | 67% (53% – 80%) | 63% (49% – 76%) |
| Specificity (95% CI) | 86% (74% – 94%) | 76% (63% – 87%) | 80% (68% – 90%) |
| PPV (95% CI) | 76% (62% – 87%) | 73% (62% – 82%) | 75% (63% – 84%) |
| NPV (95% CI) | 65% (58% – 71%) | 71% (62% – 79%) | 70% (62% – 78%) |

Legend: AUC = area under the curve, FFR = fractional flow reserve, NPV = negative predictive value, PPV = positive predictive value, RFR = resting full-cycle ratio, vFFR = vessel fractional flow reserve.

*Supplemental Table 3. Procedural characteristics (Microvascular dysfunction)*

| **Non-Culprit lesion location and characteristics, n (%)** | | | | |
| --- | --- | --- | --- | --- |
| **Variable** | **Overall  (N=111)** | **Without Microvascular Dysfunction (N=72)** | **With Microvascular Dysfunction (N=33)** | **p-value** |
| LAD | 66 (59%) | 43 (60%) | 19 (58%) | 0.836 |
| LCx | 24 (22%) | 15 (21%) | 8 (24%) | 0.695 |
| RCA | 21 (19%) | 14 (19%) | 6 (18%) | 0.878 |
| Focal disease | 27 (24%) | 21 (29%) | 4 (12%) | 0.057 |
| Long lesions (>28mm) | 44 (40%) | 26 (36%) | 15 (45%) | 0.362 |
| Bifurcation lesion | 31 (28%) | 21 (29% | 8 (24%) | 0.600 |
| Moderate or severe calcification | 17 (15%) | 12 (17%) | 4 (12%) | 0.547 |
| **3D quantitative coronary angiography (Acute-setting)** | | | | |
| **Variable** | **Overall  (N=107)** | **Without Microvascular Dysfunction (N=70)** | **With Microvascular Dysfunction (N=33)** | **p-value** |
| Minimal lumen diameter (mm), median [25^th^ – 75^th^ percentile] | 1.5 [1.3 – 1.9] | 1.6 [1.4 – 1.9] | 1.3 [1.2 – 1.7] | 0.006 |
| Diameter stenosis (%), mean ± SD | 46 ± 10 | 45 ± 10 | 48 ± 11 | 0.181 |
| Reference vessel diameter (mm), median [25^th^ – 75^th^ percentile] | 2.9 [ 2.5 – 3.3] | 2.9 [ 2.5 – 3.4] | 2.7 [ 2.4 – 3.1] | 0.091 |
| Minimal lumen area (mm^2^), median [25^th^ – 75^th^ percentile] | 1.8 [1.3 – 2.8] | 2.0 [1.5 – 2.9] | 1.4 [1.1 – 2.3] | 0.006 |
| Area stenosis (%), mean ± SD | 70 ± 11 | 68 ± 11 | 71 ± 12 | 0.213 |
| Reference vessel area (mm^2^), median [25^th^ – 75^th^ percentile] | 6.5 [5.0 – 8.5] | 6.6 [5.0 – 9.0] | 5.9 [4.4 – 7.6] | 0.094 |
| Lesion length (mm), median [25^th^ – 75^th^ percentile] | 19 [13 – 34] | 19 [12 – 34] | 21 [12 – 34] | 0.874 |
| Contour Correction (%), median [25^th^ – 75^th^ percentile] | 9 [5 – 12] | 8 [4 – 11] | 9 [4 – 13] | 0.620 |

| **Indices** | | | | |
| --- | --- | --- | --- | --- |
| **Variable** | **Overall** | **Without Microvascular Dysfunction** | **With Microvascular Dysfunction** | **p-value** |
| vFFR, (N=110) mean ± SD; median [25^th^ – 75^th^ percentile] | 0.81 ± 0.10;  0.83 [0.74 – 0.88] | N=71 0.85 [0.77 – 0.89] | N=33 0.76 [0.71 – 0.86] | 0.006 |
| FFR, (N=111) mean ± SD; median [25^th^ – 75^th^ percentile] | 0.84 ± 0.07;  0.83 [0.80 – 0.90] | N=72 0.84 [0.80 – 0.90] | N=33 0.85 [0.77 – 0.90] | 0.490 |
| RFR, (N=108) mean ± SD; median [25^th^ – 75^th^ percentile] | 0.89 ± 0.08;  0.90 [0.84 – 0.95] | N=71 0.91 [0.86 – 0.96] | N=33 0.86 [0.84 – 0.91] | 0.004 |
| Offline vFFR, (N=109) mean ± SD; median [25^th^ – 75^th^ percentile] | 0.81 ± 0.09;  0.83 [0.75 – 0.88] | N=71 0.84 [0.79 – 0.88] | N=33 0.77 [0.72 – 0.85] | 0.002 |
| vFFR ≤0.80, n (%) | 49/110 (45%) | 24 / 71 (34%) | 22 / 33 (67%) | 0.002 |
| FFR ≤0.80, n (%) | 34/111 (31%) | 20 / 72 (28%) | 12 / 33 (36%) | 0.375 |
| RFR ≤0.89, n (%) | 52/108 (48%) | 26 / 71 (37%) | 23 / 33 (70%) | 0.002 |
| Offline vFFR ≤0.80, n (%) | 44/109 (40%) | 21 / 71 (30%) | 20 / 33 (61%) | 0.003 |

Values are presented as mean ± SD, median [25^th^ – 75^th^ percentile] or n (%). Legend: 3D = three-dimensional, FFR = fractional flow reserve, LAD = left anterior descending artery, LCx = left circumflex artery, RCA = right coronary artery, RFR = resting full-cycle ratio, SD = standard deviation, vFFR = vessel fractional flow reserve.

*Supplemental Table 4. Baseline characteristics (Microvascular dysfunction)*

| **Variable** | **Overall  (N=111)** | **Without Microvascular Dysfunction (N=72)** | **With Microvascular Dysfunction (N=33)** | **p-value** |
| --- | --- | --- | --- | --- |
| Age (years), mean ± SD | 63 ± 11 | 61 ± 11 | 68 ± 11 | 0.004 |
| Male sex, n (%) | 79 (71%) | 52 (72%) | 22 (67%) | 0.562 |
| Heart rate (bpm), mean ± SD | 76 ± 15 | 77 ± 14 | 76 ± 19 | 0.891 |
| Systolic blood pressure (mmHg), mean ± SD | 121 ± 25 | 121 ± 24 | 120 ± 27 | 0.891 |
| Diastolic blood pressure (mmHg), mean ± SD | 73 ± 13 | 75 ± 13 | 69 ± 11 | 0.051 |
| **Cardiovascular risk factors, n (%)** | | | | |
| Hypertension | 52 (47%) | 35 (49%) | 13 (41%) | 0.451 |
| Dyslipidemia | 36 (32%) | 24 (34%) | 9 (29%) | 0.604 |
| Diabetes | 15 (14%) | 10 (14%) | 5 (16%) | 0.816 |
| Current smoker | 38 (34%) | 26 (36%) | 8 (26%) | 0.308 |
| Family with CAD | 49 (44%) | 31 (44%) | 16 (50%) | 0.550 |
| **Medical history and comorbidity, n (%)** | | | | |
| Prior PCI | 10 (9%) | 8 (11%) | 2 (6%) | 0.413 |
| Prior MI | 8 (7%) | 7 (10%) | 1 (3%) | 0.230 |
| Prior CVI/TIA | 4 (4%) | 2 (3%) | 2 (6%) | 0.415 |
| Prior PAD | 3 (3%) | 3 (4%) | 0 (0%) | 0.234 |
| GFR (mL/min/1.73 m²),  median [25^th^ – 75^th^ percentile] | 79 [63 – 90] | 79 [64 – 90] *(N=70) | 78 [59 – 94] *(N=32) | 0.588 |
| Haemoglobin (mmol,/L),  mean ± SD | 8.6 ± 0.9 | 8.7 ± 0.8 *(N=70) | 8.3 ± 1.2 | 0.112 |
| **Culprit lesion location, n (%)** | | | | |
| RCA | 50 (45%) | 30 (42%) | 17 (52%) | 0.346 |
| LAD | 32 (29%) | 22 (31%) | 8 (24%) | 0.506 |
| LCx | 29 (26%) | 20 (28%) | 8 (24%) | 0.704 |
| PCI culprit lesion | 111 (100%) | 72 (100%) | 33 (100%) | - |

Values are presented as mean ± SD, median [25^th^ – 75^th^ percentile] or n (%). Legend: CAD = coronary artery disease, CVI = cerebrovascular incident, GFR = glomerular filtration rate, LAD = left anterior descending artery, LCx = left circumflex artery, MI = myocardial infarction, PAD = peripheral artery disease, PCI = percutaneous coronary intervention, RCA = right coronary artery, SD = standard deviation, TIA = transient ischemic attack.

*Supplemental Figure 1.* Bland-Altman plot of differences against the means of a) vFFR and FFR with mean bias ± SD of -0.03 ± 0.09 and median error [25^th^ – 75^th^ percentile] of -0.02 [-0.08 – 0.02], b) vFFR and RFR with mean bias ± SD of -0.08 ± 0.11 and median error of -0.07 [-0.13 – -0.02], and c) FFR and RFR with mean bias ± SD of -0.05 ± 0.06 and median error of -0.05 [-0.08 – -0.02]. Legend: The mean bias is represented by the dashed grey line and the 95% limits of agreement (mean bias ± 1.96*SD) are represented by the dashed red lines. FFR = fractional flow reserve, RFR = resting full-cycle ratio, vFFR = vessel fractional flow reserve.

| Scatterplot | Bland-Altman plot |
| --- | --- |
|  |  |
|  |  |
|  |  |

*Supplemental Figure 2.* ROC-curve for vFFR, RFR and offline vFFR compared to FFR.

Legend: AUC = area under the ROC curve, FFR = fractional flow reserve, ROC = Receiver operating characteristic, RFR = resting full-cycle ratio, vFFR = vessel fractional flow reserve.

*Supplemental Figure 3.* Comparison of vFFR, FFR, and RFR measurements in patients with and without microvascular dysfunction (CFR<2.5 and IMR≥25). Boxplots show the distribution of vFFR, FFR, and RFR values, with medians and p-values for group comparisons. FFR = fractional flow reserve, RFR = resting full-cycle ratio, vFFR = vessel fractional flow reserve.

Microvascular dysfunction (CFR<2.5 and IMR≥25) was present in 41 patients (39%). In patients with microvascular dysfunction, median [25^th^ – 75^th^ percentile] vFFR was 0.77 [0.72 – 0.86], FFR was 0.83 [0.77 – 0.90], and RFR was 0.87 [0.84 – 0.93], while in patients without microvascular dysfunction, median [25^th^ – 75^th^ percentile] vFFR was 0.85 [0.77 – 0.89], FFR was 0.84 [0.80 – 0.91], and RFR was 0.91 [0.86 – 0.96] (**Figure S3**). Median vFFR was significantly lower as compared to FFR in patients with microvascular dysfunction (p<0.001), but not in patients without microvascular dysfunction (p=0279) (**Figure S3**). The median [25^th^ – 75^th^ percentile] vFFR-FFR difference in patients with vs. without microvascular dysfunction was -0.05 [-0.11 – 0.00] vs. 0.00 [-0.05 – 0.03] (p=0.004).
